# Research progress on the multidimensional mechanisms underlying impaired spermatogenesis induced by pathological microenvironment remodeling in chronic prostatitis

**DOI:** 10.3389/fimmu.2026.1793156

**Published:** 2026-03-11

**Authors:** Jing Li, Zhipeng Jiang, Wen Luo, Kaihua Tang, Decan Liang, Xijian Luo, Lei Liu, Zongmin Long, Hui Huang, Weiwei Chen, Yichi Zhang

**Affiliations:** Department of Urology, The Third Affiliated Hospital of Zunyi Medical University (The First People’s Hospital of Zunyi), Zunyi, China

**Keywords:** Chronic prostatitis, immune cells, microenvironment, oxidative stress, sperm, sperm DNA fragmentation

## Abstract

Chronic Prostatitis/Chronic Pelvic Pain Syndrome (CP/CPPS), a prevalent urological and andrological condition among men of reproductive age, induces persistent pathological alterations. These alterations remodel the microenvironment of the prostate and reproductive tract through multiple pathways, thereby severely impairing sperm spermatogenesis, maturation, and function. By constructing a multidimensional interaction network encompassing “inflammation-oxidation-endocrine-microbiota, “ this article elucidates the four core pathological mechanisms by which the CP/CPPS microenvironment damages the full cycle of sperm development: (1) local inflammatory storms and immune cell infiltration hindering sperm development; (2) the collapse of the antioxidant defense system due to oxidative stress imbalance and excessive reactive oxygen species (ROS) generation; (3) metabolic homeostasis disruption in the spermatogenic microenvironment caused by neuroendocrine and biochemical disorders; and (4) sperm functional impairment resulting from heterogeneous alterations in the reproductive tract and gut microbiomes. This review systematically reveals the cascading impact of CP/CPPS on the entire chain of “testicular spermatogenesis–epididymal maturation–fertilization capacitation.” Furthermore, it posits that future research should focus on multi-omics mechanism resolution and shift towards a multi-target, precision combination intervention strategy of “anti-inflammation, antioxidation, endocrine regulation, and microecological reconstruction, “ providing a theoretical basis and translational direction for improving clinical reproductive outcomes in patients.

## 1 Introduction

Chronic Prostatitis/Chronic Pelvic Pain Syndrome (CP/CPPS) is a prevalent condition in andrology, characterized by persistent pain or discomfort in the pelvic region for ≥3 months. It is often accompanied by lower urinary tract symptoms (LUTS), such as urinary frequency and urgency, as well as sexual dysfunction, including ejaculatory pain. Epidemiological studies indicate that the lifetime prevalence of prostatitis-like symptoms in men ranges from 35% to 50%, with an annual prevalence of approximately 8%–8.2%, being particularly common in men under 50 years of age (1–3). According to the National Institutes of Health (NIH) classification system, prostatitis is categorized into four types: Type I (acute bacterial prostatitis), Type II (chronic bacterial prostatitis), Type III (CP/CPPS, subdivided into IIIa inflammatory and IIIb non-inflammatory), and Type IV (asymptomatic inflammatory prostatitis). Among these, CP/CPPS accounts for over 90% of all chronic prostatitis cases (4, 5). Its pathogenesis involves a multitude of pathological processes, including infection (encompassing occult infection), autoimmunity, neuroendocrine dysregulation, pelvic floor dysfunction, gut microbiota dysbiosis, and psychosocial factors (4, 5). Due to its complex etiology and the lack of a gold-standard diagnostic method or uniformly effective treatment protocols, CP/CPPS remains a significant challenge in both clinical practice and basic research. Given the complexity of its etiology and the absence of specific “gold standard” diagnostic tools, CP/CPPS continues to be a thorny issue in clinical diagnosis and fundamental research.

In this context, accurately defining the disease phenotype of study subjects is crucial for elucidating pathological mechanisms. Clinicians must first distinguish inflammatory diseases with similar symptoms but distinct mechanisms to avoid confounding caused by “phenotypic mimicry” (same symptoms, different diseases). For example, Granulomatous Prostatitis (GP) clinically mimics CP/CPPS or even prostate cancer, but is essentially a granulomatous inflammatory response involving specific infections or iatrogenic factors (6). Failure to distinguish these heterogeneous lesions will severely interfere with the analysis of CP/CPPS-specific microenvironmental alterations. Furthermore, given that CP/CPPS (NIH Type III) is essentially a “diagnosis of exclusion, “ its symptom and disease profiles exhibit significant heterogeneity. In clinical cohort studies, besides strictly distinguishing Type II chronic bacterial prostatitis (CBP) from Type III CP/CPPS, it is necessary to exclude other functional disorders that may cause similar pelvic pain and LUTS. Therefore, only by establishing strict classification boundaries and differential diagnosis protocols can we accurately explore the “prostate microenvironment remodeling–spermatogenesis impairment” link under a homogenized research framework, thereby ensuring the robustness of mechanistic inferences and the comparability of clinical translation.

**Figure 1:**
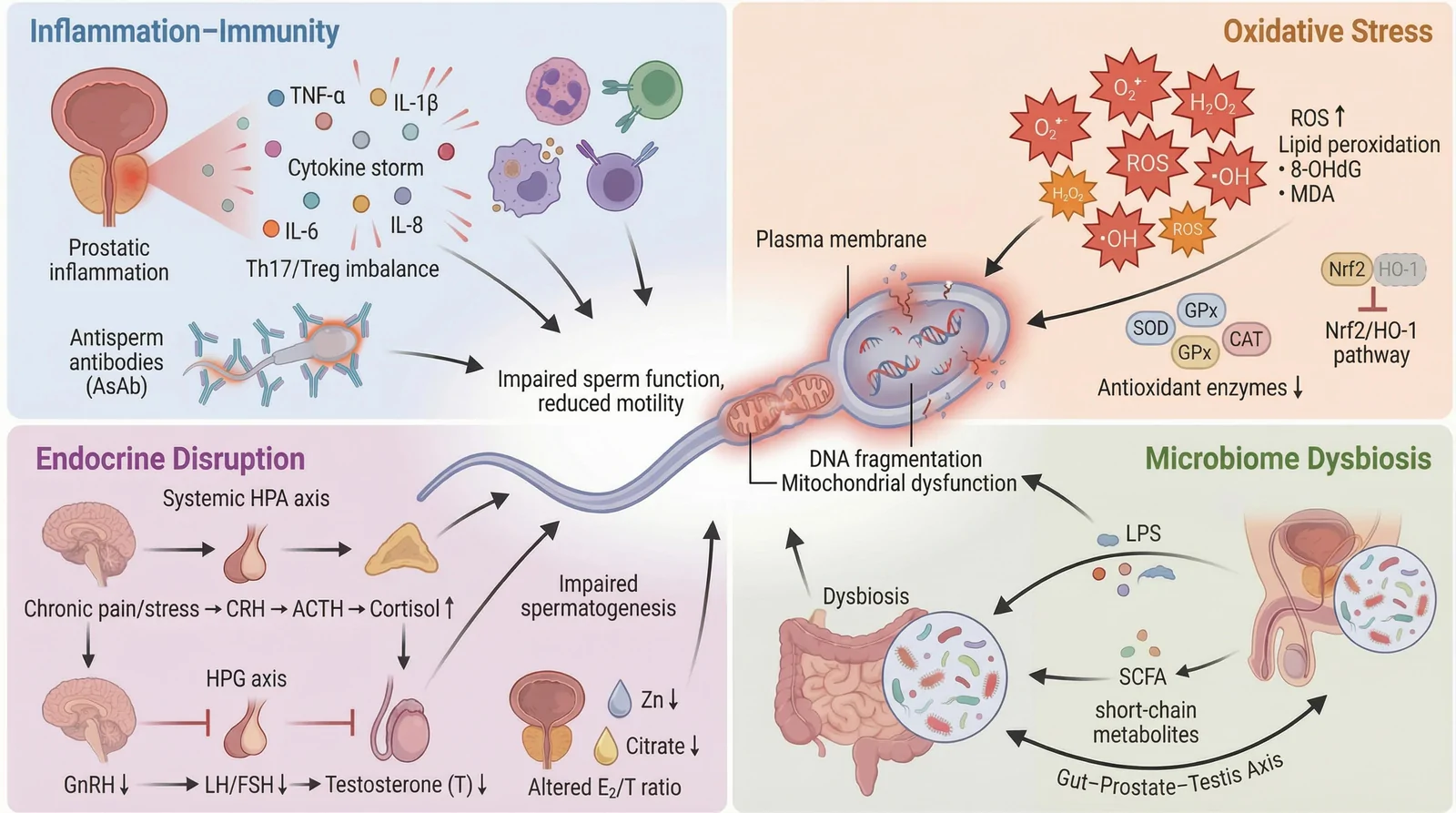
The four-dimensional interactive pathogenic network of the CP/CPPS microenvironment impairing the full life cycle of sperm.

Increasing evidence indicates that the impact of CP/CPPS extends beyond the urinary system, having significant implications for male reproductive health, particularly concerning the risk of infertility. The prevalence of CP/CPPS in the general male population is approximately 2.2%–9.7% (median ~8%), whereas its detection rate is significantly elevated among infertile men (reported in some studies as 20%–39%), suggesting a robust correlation between the two (1, 5, 7). This correlation is evidenced not only by prevalence rates but also by alterations in semen parameters. Studies have demonstrated that patients with CP/CPPS often exhibit a spectrum of semen abnormalities, including decreased sperm concentration and total count, reduced sperm viability and progressive motility, lowered motility grades, and prolonged semen liquefaction time (8, 9). Persistent prostatic inflammation can involve accessory glands, such as the seminal vesicles and epididymis, and induce systemic low-grade inflammation, thereby interfering with testicular spermatogenesis as well as sperm maturation, storage, and transport within the reproductive tract. Current research posits that CP/CPPS-related infertility primarily involves multiple mechanisms: secretory dysfunction of the prostate and accessory glands, abnormalities in local and systemic immune/inflammatory responses, excessive generation of reactive oxygen species (ROS) leading to oxidative stress injury, and secondary impairment of sperm DNA structure and integrity (8). However, current evidence regarding “how the CP/CPPS-induced disorder of the prostate and reproductive tract microenvironment affects spermatogenesis, maturation, and function across multiple dimensions” remains fragmented. Most studies focus on isolated risk factors or clinical phenotypes, and basic research is predominantly cross-sectional or limited to small sample cohorts, lacking a systematic synthesis of the interactions and causal chains among various pathological mechanisms. In particular, previous literature has largely focused on alterations in semen parameters while neglecting the cascading pathological changes in the upstream testicular spermatogenic microenvironment and the midstream epididymal maturation microenvironment. Therefore, this review aims to explore the four core pathological mechanisms by which the microenvironment impairs the full cycle of sperm development: local inflammatory storms and immune cells, oxidative stress imbalance, neuroendocrine and biochemical dysregulation, and the heterogeneity of the reproductive and gut microbiomes. By integrating evidence on the impact of CP/CPPS-related prostate microenvironmental remodeling on sperm development and semen quality, this article seeks to deepen the understanding of the nature of CP/CPPS-associated infertility and provide a theoretical basis and translational direction for future mechanistic research and precision intervention strategies (**Figure 1**).

This diagram illustrates how CP/CPPS disrupts sperm development and function through a complex network involving Inflammation-Immunity, Oxidative Stress, Endocrine Disruption, and Microbiome Dysbiosis (1). Inflammation-Immunity (Top Left): CP/CPPS triggers a local and systemic “cytokine storm” characterized by elevated pro-inflammatory cytokines (TNF-α, IL-1β, IL-6, IL-8), immune cell infiltration, Th17/Treg imbalance, and the production of antisperm antibodies (AsAb). (2) Oxidative Stress (Top Right): Inflammation-driven excessive reactive oxygen species (ROS) generation and the collapse of antioxidant defense systems (e.g., SOD, CAT, GPx, and the Nrf2/HO-1 pathway) lead to lipid peroxidation and the accumulation of oxidative damage markers (MDA, 8-OHdG). (3) Endocrine Disruption (Bottom Left): Chronic pain/stress activates the systemic HPA axis (Cortisol ↑), inhibiting the HPG axis (GnRH, LH/FSH, Testosterone ↓). This is compounded by prostatic secretory dysfunction (decreased Zn and Citrate) and an altered local Estradiol/Testosterone (E2/T) ratio. (4) Microbiome Dysbiosis (Bottom Right): Dysbiosis of the gut and genitourinary microbiota amplifies inflammation and metabolic disruption via the “Gut-Prostate-Testis Axis, “ marked by increased LPS/PAMPs and reduced short-chain fatty acids (SCFAs). Collectively, these pathways converge to cause sperm mitochondrial dysfunction, increased DNA fragmentation, and reduced motility.

## 2 Overview of sperm development and microenvironmental regulation in chronic prostatitis

Spermatogenesis and sperm development represent a multi-stage process that proceeds systematically along the intricate axis formed by the testis, epididymis, and male accessory glands, highly dependent on a stable local microenvironment and normal secretory function. As a common inflammatory condition of the male accessory glands, CP/CPPS acts as a core pathological factor interfering with this process. It not only directly causes dysfunction in prostatic secretion but also, through a mechanism of “serial involvement, “ facilitates the retrograde diffusion of the inflammatory microenvironment along the reproductive tract. This impairs the secretory functions of multiple segments, including the seminal vesicles and epididymis, resulting in a comprehensive deterioration of seminal plasma composition. Concurrently, the immune–inflammatory storm, oxidative stress imbalance, and systemic endocrine disruption triggered by CP/CPPS directly attack spermatozoa and breach protective structures such as the blood–testis barrier. This disrupts the homeostasis of the spermatogenic microenvironment at both local and systemic levels, ultimately causing multi-dimensional, full-chain impairment to sperm genesis, maturation, motility, and genetic integrity.

Spermatogenesis is the core process of male germ cell development, proceeding in highly ordered stages within the seminiferous tubules of the testis. This process initiates with the committed differentiation of spermatogonial stem cells (SSCs) and sequentially undergoes spermatogonial mitotic expansion, spermatocyte meiosis, and spermiogenesis (morphological construction), finally forming mature spermatozoa with motility and fertilization capacity. SSCs possess both self-renewal and differentiation capabilities; differentiated spermatogonia undergo mitotic expansion to form spermatocytes, which complete two meiotic divisions to form haploid spermatids. Subsequently, during spermiogenesis, these spermatids undergo chromatin remodeling and the structural perfection of the flagellum and mitochondria, ultimately generating structurally mature spermatozoa. Newborn sperm require further functional maturation within the epididymis to acquire fertilization capability. The complete human spermatogenic cycle takes approximately 60–75 days, with certain individual variations (10–13).

During sperm development, the testis, epididymis, vas deferens, ejaculatory duct, seminal vesicles, and prostate constitute a continuous “spermatogenesis–transport–ejaculation” axis. After generation in the seminiferous tubules, sperm pass sequentially through the epididymis and vas deferens, mixing with secretions from the seminal vesicles and prostate in the ejaculatory duct to form seminal plasma. The seminal vesicles primarily provide energy substrates such as fructose and participate in semen coagulation/liquefaction, while prostatic fluid regulates plasma pH, capacitation, and the acrosome reaction via zinc, citrate, and various proteases, which is crucial for sperm function (14, 15). Previous studies indicate that so-called male accessory gland infection (MAGI), occurring in the prostate, seminal vesicles, vas deferens, and epididymis, typically disseminates retrogradely via the “urethra–prostate–seminal vesicle–vas deferens–epididymis–testis” lumen, potentially impairing the secretory function of multiple accessory gland segments simultaneously (16–21). CP/CPPS is the most common phenotype of MAGI; its associated infection/inflammation has been confirmed to lead to decreased semen volume and reduced levels of neutral α-glucosidase, fructose, and zinc in seminal plasma, accompanied by elevated pH. This reflects the “serial involvement” of the epididymis, seminal vesicles, and prostate in terms of function (5), providing an anatomical and pathophysiological basis for the amplification of microenvironmental disorders caused by the prostate along the entire reproductive tract, thereby interfering with multiple links in sperm development.

From the perspective of microenvironmental regulation, Sertoli cells provide immune privilege and metabolic support for meiosis and spermatogenesis by constructing the blood–testis barrier, while Leydig cells in the interstitium maintain the endocrine homeostasis of the spermatogenic microenvironment via testosterone synthesis. Additionally, peritubular myoid cells/pericytes participate in local microenvironmental regulation alongside immune cells (22, 23). This complex process involves precise intercellular communication, hormonal cascades, mitochondrial energy metabolism, and chromatin remodeling, exhibiting high sensitivity to redox balance, cytokine networks, and local hormone gradients. In pathological states, interfering factors such as inflammatory cytokine storms, reactive oxygen species (ROS) accumulation, hormonal fluctuations, and microbial metabolites can disrupt the homeostatic balance of the spermatogenic process, leading to spermatogenic arrest or functional defects (23).

The mechanism by which CP impairs male reproductive function is characterized by multi-dimensional and multi-systemic pathology. Its core pathological network is formed by the interaction of immune–inflammatory cascades, oxidative stress imbalance, endocrine dysregulation, and microbiome dysbiosis. Prospective clinical studies indicate that patients with CP/CPPS exhibit significant impairment in multiple semen quality parameters compared to healthy males, manifesting as declines in total/progressive motility, normal morphology rates, and sperm viability, accompanied by an elevated sperm DNA fragmentation index (DFI) (24). These changes primarily stem from alterations in seminal plasma composition caused by abnormal prostatic secretion, which subsequently exerts direct toxic effects on post-testicular sperm. Molecularly, the positive feedback loop formed by inflammatory signaling pathways (e.g., NF-κB/NLRP3) and ROS triggers lipid peroxidation, protein carbonylation, and oxidative nucleic acid damage such as 8-hydroxy-2’-deoxyguanosine (8-OHdG) (25–33). These molecular events establish a critical link between the prostatic inflammatory microenvironment and sperm dysfunction by destroying sperm plasma membrane integrity, mitochondrial electron transport chain function, and chromatin stability. Research suggests that the Nrf2-mediated antioxidant defense system serves as an important antagonistic mechanism in this pathological process and is regarded as a potential therapeutic target (34). Furthermore, chronic inflammation and oxidative stress alter the permeability of the blood–testis barrier and the epididymal epithelial barrier, continuously exposing germ cells at various developmental stages (including spermatogenesis, maturation, and functional acquisition) to circulating inflammatory mediators and immune attacks. These factors ultimately lead to multi-link impairment of key biological processes such as spermatogenesis, epididymal maturation, and fertilization capacity (35, 36).

Beyond direct damage to the local microenvironment, CP/CPPS exerts remote negative regulatory effects via systemic pathways. The overactivation of the hypothalamic–pituitary–adrenal (HPA) axis induced by chronic pain can interfere with GnRH pulses and inhibit testosterone synthase activity, exacerbating metabolic disturbances in the spermatogenic microenvironment. Concurrently, the heterogeneous alteration of the prostate–urinary–gut microbiota constitutes a new pathogenic dimension: dysbiotic flora not only directly activate innate immunity by releasing pathogen-associated molecular patterns (PAMPs) like lipopolysaccharides (LPS) but also remodel the host antioxidant defense system by interfering with the metabolism of short-chain fatty acids, bile acids, and steroid hormones. This exerts a critical pathological amplification effect within the “microbiota–metabolism–immune–endocrine” four-dimensional interaction network, ultimately leading to the comprehensive deterioration of semen parameters.

## 3 The inflammatory-immune microenvironment: the “storm center” of sperm development

The core pathological feature of Chronic Prostatitis (CP) lies in the persistent local inflammatory response and severe imbalance of immune homeostasis within the lesion area. This pathological alteration is not confined to the prostate gland itself but diffuses throughout the reproductive tract via high concentrations of inflammatory mediators in secretions (particularly TNF-α, IL-1β, IL-6, and IL-8), forming a widespread “inflammatory microenvironment.” Although the prostate is typically regarded as the primary inflammatory focus in CP/CPPS, relevant inflammatory mediators and the secondary immune responses they induce are believed to involve the testis and epididymis to a certain extent. This leads to multi-segmental impairment of spermatogenic and sperm transport functions, constituting a multi-organ pathological network that globally affects sperm genesis and maturation (21, 37). At the molecular level, this process is typically initiated by pathogen-associated molecular patterns (PAMPs) and endogenous damage-associated molecular patterns (DAMPs). These patterns activate the nuclear transcription factor NF-κB via the TLR2/4–MyD88 signaling pathway, subsequently upregulating the expression of various pro-inflammatory cytokines. These factors do not act in isolation; rather, they induce the assembly of the NLRP3 inflammasome, further amplifying the cascading release of IL-1β and IL-18, thereby constructing a self-amplifying “pro-inflammatory–oxidative” positive feedback loop. Persistent activation of the NF-κB/NLRP3 pathway not only significantly upregulates the generation of reactive oxygen species (ROS), triggering mitochondria-caspase-dependent apoptosis, but also severely disrupts cellular autophagy and energy metabolism, ultimately leading to decreased survival rates and impaired function of germ cells (25–27, 38–40). This immune storm driven by the cytokine network constitutes the molecular basis for impaired sperm development. The following sections will systematically elucidate the cascading damage of this inflammatory network on the full cycle of sperm development from four dimensions: molecular mechanism drivers, disruption of the testicular spermatogenic barrier, remodeling of the epididymal maturation environment, and immune attack within the seminal fluid.

### 3.1 Remote impairment of the testicular spermatogenic microenvironment: barrier disruption and cytotoxicity

The smooth progression of spermatogenesis relies heavily on the unique local “immune privilege” status of the testis and a finely regulated hormonal microenvironment, while the inflammatory storm associated with CP severely threatens this homeostasis by disrupting the blood–testis barrier (BTB). First, at the structural level, inflammatory cytokines (e.g., TNF-α, TGF-β3, IL-1α) attack the molecular scaffold of the BTB, causing the internalization, degradation, or aberrant localization of key tight junction proteins (claudin-11, occludin, ZO-1), directly leading to the collapse of the barrier structure (11, 41–43). Once the integrity of the BTB is lost, dual adverse consequences ensue: on one hand, germ cells in the meiotic or earlier stages lose critical immune shelter, becoming directly exposed to circulating inflammatory mediators and the attacks of infiltrating Th1/Th17 cells and M1 macrophages (5, 24); on the other hand, high concentrations of TNF-α and HMGB1 can activate the mitochondrial apoptotic pathway (manifested as Bcl-2 downregulation, Bax upregulation, and Caspase-3 activation), directly triggering programmed cell death in spermatogonial stem cells (SSCs) and other germ cells at various stages (44, 45). Second, inflammation interferes with the spermatogenic regulatory network at the functional level. This pro-inflammatory environment can directly activate NF-κB and MAPK signaling pathways within SSCs, leading to cell cycle arrest, DNA damage, and p53-dependent apoptosis (46–48). It can also induce endoplasmic reticulum stress (ERS) via the TLR4/NF-κB pathway, further aggravating SSC apoptosis and dysfunction (46). Furthermore, persistently elevated IL-6 has been confirmed to inhibit DNA synthesis in spermatocytes and arrest their meiotic progression, indirectly leading to the exhaustion of SSC differentiation potential (49–51). Concurrently, the inflammatory microenvironment further deteriorates the overall spermatogenic microenvironment by inhibiting the steroidogenic function of Leydig cells (e.g., TNF-α inhibits the activity of key testosterone synthesis enzymes) and weakening the nutritional and physical support provided by Sertoli cells to germ cells (44, 52). Notably, local prostatic inflammation may diffuse to the testis via paracrine mechanisms (e.g., the IL-1RA–AR axis), indirectly interfering with spermatogenesis (53, 54). Relevant animal studies also indicate that the infiltration and accumulation of CP-induced inflammatory cytokines within the testis serve as a key upstream mechanism leading to oligozoospermia and male fertility decline (45, 55).

### 3.2 Interference with epididymal sperm maturation: environmental remodeling and impaired molecular transfer

The epididymis is not merely an organ for sperm storage but a critical site for the acquisition of motility and fertilization capacity. This process is highly dependent on the secretory activity of epididymal epithelial cells and molecular transfer mediated by epididymosomes. However, the inflammatory state induced by CP profoundly remodels the microenvironment within the epididymal lumen. On one hand, inflammation alters the secretory profile of the epididymal epithelium and the composition of exosomes (including functional proteins, lipids, and small RNAs), preventing sperm from acquiring correct membrane protein modifications during maturation, altering glycosylation patterns, and even causing the loss of fertilization-related receptors (56–58). On the other hand, inflammation disrupts the physicochemical properties within the epididymal lumen, including redox balance, pH homeostasis, and Ca²^+^/HCO_3_⁻ ion equilibrium. These changes in environmental factors directly affect the fluidity of sperm membrane lipids and flagellar beating function. Relevant clinical studies indicate a significant correlation between elevated levels of inflammatory cytokines in the seminal plasma of CP patients and sperm maturation disorders as well as functional defects (59, 60). In summary, by interfering with epididymal epithelium–sperm interaction and exosome transfer, inflammation reduces the supply of maturation factors and causes qualitative abnormalities, thereby laying the foundation for subsequent declines in capacitation and motility (61, 62).

### 3.3 Multidimensional impairment of sperm function, morphology, and genetic material

**Figure 2:**
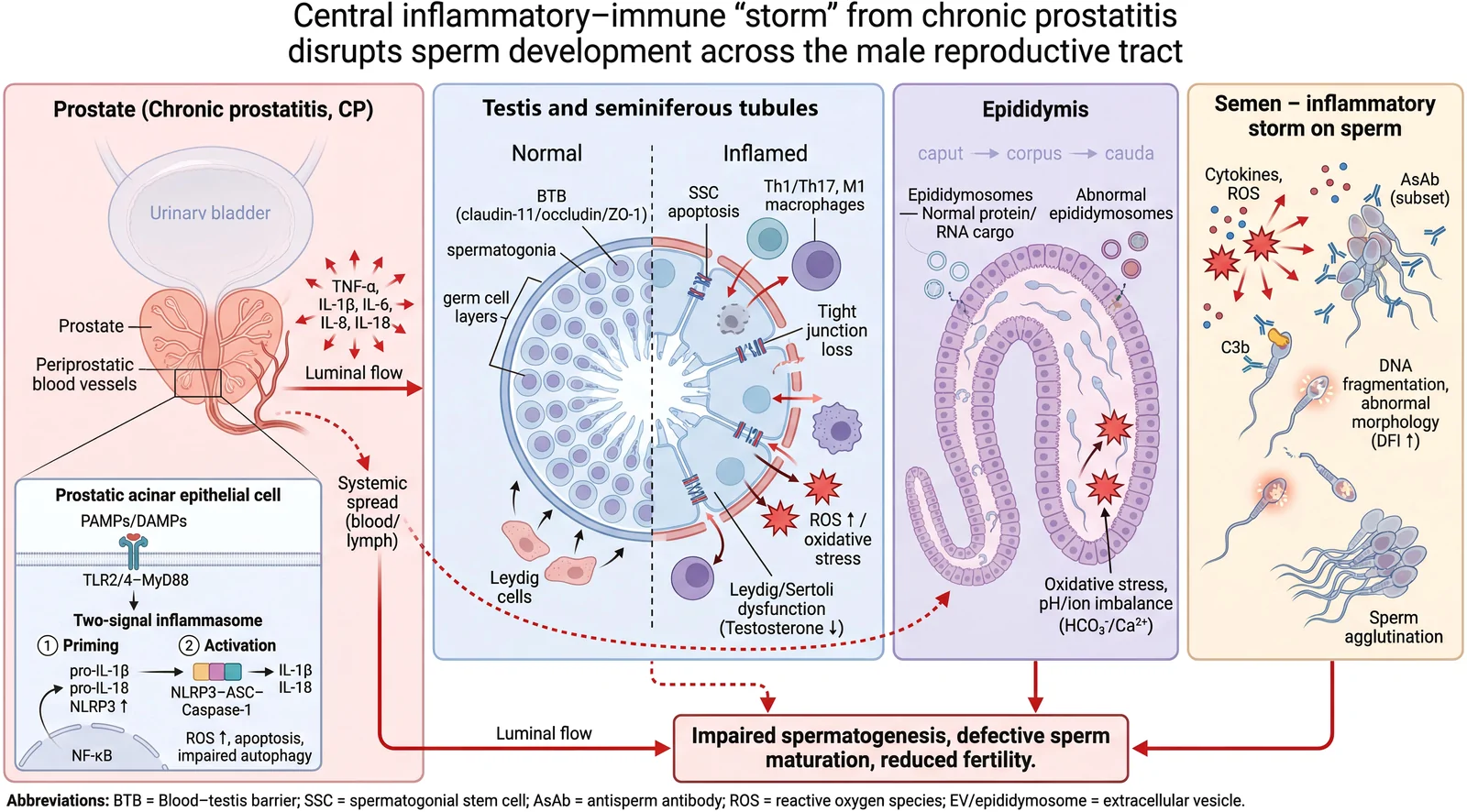
Schematic illustration of the inflammation–immune “storm” triggered CP/CPPS and its detrimental effects on spermatogenesis and sperm maturation.

When sperm finally enter the seminal environment, they are directly exposed to the forefront of the “immune–inflammatory” storm, facing multiple strikes from anti-sperm antibodies (AsAb), cytokine toxicity, and oxidative stress. **First, immune attack and antibody formation:** The disruption of the BTB exposes sequestered sperm antigens, inducing B cell activation and AsAb production. Systematic reviews and meta-analyses show that the positive rate of AsAb in CP patients is significantly higher than in healthy controls (63); a study by Jiang et al. reported a pooled odds ratio (OR) as high as 3.26 (64). Once formed, anti-sperm antibodies can bind to the sperm surface, leading to decreased sperm motility and affecting sperm–oocyte interaction; concurrently, they can interfere with key fertilization steps such as capacitation and the acrosome reaction. Some antibodies can also activate the complement cascade, causing sperm membrane damage or even lysis, thereby impairing sperm fertilization function through multiple pathways (65, 66). **Second, direct inhibitory effects of cytokines:** When the levels of pro-inflammatory cytokines (represented by TNF-α) in seminal plasma rise to a certain threshold, they can exert direct toxic inhibition on sperm, manifested as declines in sperm vitality and progressive motility. Potential mechanisms include damaging mitochondrial energy metabolism and inducing excessive generation of nitric oxide/reactive nitrogen species (NO/RNS), thereby reducing flagellar movement efficiency. Furthermore, inflammation-associated elevations in oxidative/nitrosative stress may interfere with capacitation-related signaling pathways, subsequently affecting the sperm capacitation process (34, 67). Similarly, IL-6 significantly reduces progressive motility, with mechanisms likely related to excessive NO production or triggering neutrophils to release hypochlorous acid that damages the sperm membrane, also exhibiting a dose-dependent inhibitory effect (68). Meanwhile, IL-8, as a potent chemokine, indirectly destroys sperm structure by recruiting neutrophils to release large amounts of ROS and hydrolases (54). **Third, genetic material and morphological damage:** The inflammatory environment is a significant inducer of sperm morphological abnormalities and DNA damage. Research data indicate that patients with CP/CPPS often exhibit a significantly reduced proportion of morphologically normal sperm and an abnormally elevated sperm DNA fragmentation index (DFI) (with some studies reporting a median of 16.0%, nearly double that of the control group at 8.4%) (24). Sperm DNA fragmentation is significantly negatively correlated with major semen parameters and the tropomyosin 1/2 mRNA ratio (24). Oxidative stress and apoptotic mechanisms induced by factors such as TNF-α not only adversely affect sperm vitality and morphology but also directly or indirectly lead to DNA damage (9). In summary, the chronic inflammatory state may also lead to abnormal sperm chromatin condensation, manifested as increased high DNA stainability (HDS). This occult damage at the genetic material level not only significantly lowers the natural conception rate but may also have long-term negative impacts on the early developmental potential of the embryo (**Figure 2**).

In CP lesions, prostatic acinar epithelial cells stimulated by PAMPs/DAMPs activate NF-κB via TLR2/4–MyD88 signaling and, through the inflammasome “two-signal” process, promote the assembly and activation of the NLRP3 inflammasome, thereby amplifying the cascade release of inflammatory mediators such as IL-1β and IL-18 (along with TNF-α, IL-6, and IL-8). This results in elevated ROS, increased apoptosis, and impaired autophagy. Inflammatory mediators can spread downstream with the intraluminal flow of prostatic fluid and disseminate systemically through the blood/lymphatic circulation. In the testis, inflammation and oxidative stress disrupt the blood–testis barrier (BTB; dysregulation of tight-junction proteins such as claudin-11, occludin, and ZO-1), promote infiltration of Th1/Th17 cells and M1 macrophages, and induce spermatogonial stem cell (SSC) apoptosis; meanwhile, they cause Leydig/Sertoli cell dysfunction and reduced testosterone. In the epididymis, oxidative stress and disturbed pH/ion homeostasis (HCO3−/Ca2+) alter the transport and delivery of protein/RNA cargo in epididymosomes, interfering with terminal sperm maturation. In semen, immune assaults involving cytokines/ROS, antisperm antibodies (AsAb), and complement (e.g., C3b) can lead to sperm agglutination, DNA fragmentation, and abnormal morphology (increased DFI), ultimately resulting in impaired spermatogenesis, defective sperm maturation, and reduced fertility.

## 4 Oxidative stress: a key feature of the CP microenvironment

Oxidative stress represents one of the most destructive core pathological features within the microenvironment of Chronic Prostatitis (CP). Its essence lies in the severe imbalance between the excessive generation of reactive oxygen species (ROS) and the antioxidant defense system, a disequilibrium that plays a pivotal driving role in the onset and chronicity of CP. Notably, ROS play a subtle “double-edged sword” role in male reproductive physiology: while physiological levels of ROS are essential signaling molecules for normal fertilization processes such as sperm capacitation, hyperactivation, and the acrosome reaction (69–71), in the pathological microenvironment of CP, this delicate balance is thoroughly disrupted. Excessive ROS transform into cytotoxic factors, causing significant and irreversible damage to the fine structure and function of spermatozoa (32, 33, 70, 72–76). Clinical epidemiological data further confirm this hazard: approximately 30% to 80% of male infertility cases are confirmed to be closely associated with ROS-induced sperm injury (77). Biochemical indicators from semen analysis also show that ROS levels in the semen of CP patients are significantly higher than in healthy controls, while the Total Antioxidant Capacity (TAC) and related enzyme activities, which reflect defense capabilities, exhibit a significant decline (78, 79). The persistence of this oxidative stress state directly leads to decreased sperm concentration, reduced motility, and an elevated rate of morphological abnormalities, thereby significantly diminishing the natural conception rate in men (73).

### 4.1 Imbalance of the oxidative-antioxidant system: multi-source generation and collapse of defense barriers

In the pathological microenvironment of CP, the formation of oxidative stress stems from the dual pathological mechanisms of “enhanced attack” and “failed defense, “ which jointly drive the deterioration of the microenvironment. The generation of ROS presents a multi-source eruptive pattern, primarily involving three intertwined mechanisms: First, the inflammatory response activates infiltrating neutrophils and macrophages, triggering a violent “respiratory burst” that produces massive amounts of superoxide anions via NADPH oxidase (NOX), constituting the primary source of local ROS (80). Second, inflammation causes damage to the prostate epithelium and accessory gland cells, inducing mitochondrial dysfunction and electron transport chain leakage. This not only leads to a significant increase in ROS generation but also further amplifies the local inflammatory response by releasing pro-inflammatory signals (81). Finally, high concentrations of pro-inflammatory cytokines (e.g., IL-1β, TNF-α) further exacerbate ROS accumulation by upregulating the expression of intracellular oxidases (including NOX) and directly disrupting mitochondrial homeostasis (82, 83). These three mechanisms promote each other, forming a vicious “pro-inflammatory–oxidative” positive feedback loop: inflammatory cells induce ROS production, while ROS, in turn, activate pro-inflammatory transcriptional pathways such as NF-κB and the NLRP3 inflammasome, further sustaining the inflammatory state (28, 29).

Concurrently, the antioxidant defense system undergoes a comprehensive collapse, rendering it unable to effectively scavenge excessive ROS. Studies have found that in the prostatic fluid and semen of CP patients, the activities of key antioxidant enzymes—such as Superoxide Dismutase (SOD), Catalase (CAT), and Glutathione Peroxidase (GPx)—are significantly reduced. Meanwhile, levels of markers reflecting oxidative damage (such as MDA and 8-OHdG) are significantly elevated, directly confirming the severe destruction of the antioxidant barrier (84). Further analysis reveals that the decline in Total Antioxidant Capacity (TAC) and the elevation of lipid peroxidation products (such as 8-IsoP) in expressed prostatic secretions (EPS) are not only closely related to bacterial culture positivity and inflammatory phenotypes but are also positively correlated with the degree of sperm DNA damage, suggesting that the imbalance of the local biochemical environment is the direct cause of impaired reproductive function (78). Although definitive conclusions from large-scale randomized controlled trials are currently lacking, small-sample clinical and animal studies have indicated that exogenous antioxidant interventions (e.g., supplementation with Vitamin C/E, N-acetylcysteine, CoQ10) can, to a certain extent, reconstruct the oxidative balance and improve select biological indicators (85).

### 4.2 Structural susceptibility of sperm to oxidative stress and mechanisms of molecular damage

Due to their unique biological structure, spermatozoa are the primary targets of oxidative stress, exhibiting extremely high vulnerability. Their cell membranes are rich in polyunsaturated fatty acids (PUFAs) that are highly susceptible to oxidation, and due to the minimal volume of cytoplasm, they lack sufficient intracellular antioxidant enzyme reserves and buffering capacity. Furthermore, sperm lack the comprehensive DNA repair mechanisms possessed by somatic cells (86–88), making them highly prone to multi-dimensional molecular attacks within the CP microenvironment. From the perspective of the overall mechanistic chain, oxidative stress damage to sperm typically presents a cascading characteristic of “exterior-to-interior, structure-to-function”: membrane lipids are damaged first, followed by amplified perturbations in metabolism and mitochondrial homeostasis, ultimately involving genetic material and key protein functions.

Among the multitude of molecular attacks, membrane damage caused by lipid peroxidation occurs first. ROS attack PUFAs on the membrane, triggering a chain peroxidation reaction that generates cytotoxic aldehydic products such as malondialdehyde (MDA) and 4-hydroxynonenal (4-HNE) (30, 89). This process destroys the integrity of the phospholipid bilayer, leading to a loss of membrane fluidity and increased permeability. The destruction of membrane structure not only directly weakens sperm motility but also severely interferes with the ability of sperm to fuse with the oocyte plasma membrane during fertilization (sperm–oocyte fusion) (30, 34, 90); meanwhile, membrane hardening also hinders the sperm acrosome reaction, a critical step for penetrating the zona pellucida (34). It should be noted that the impact of lipid peroxidation may not be limited to the “sperm membrane itself”: its reaction products (e.g., MDA, 4-HNE) can form adducts with proteins and further interfere with mitochondrial function and energy metabolism, thereby forming a self-amplifying oxidative–metabolic vicious cycle, creating conditions for subsequent damage at the genetic and protein levels. Concurrently, evidence from other studies on spermatogenic disorders suggests that metabolic disorders in testicular interstitial Leydig cells may be related to lipid peroxidation/abnormal lipid metabolism and may be accompanied by an imbalance in INSL3–Androgen Receptor (AR) signaling. Under certain conditions, this could weaken local steroidogenesis and androgen support, thereby indirectly amplifying spermatogenesis and maturation disorders via “endocrine microenvironment imbalance” (91, 92).

Next is Sperm DNA Fragmentation (SDF). Against the backdrop of altered membrane permeability and metabolic amplification effects, ROS (particularly the highly reactive hydroxyl radical, ·OH) are more likely to accumulate in the perinuclear microenvironment and penetrate the nuclear envelope, directly attacking sperm DNA. This induces oxidative base modifications (such as the formation of 8-OHdG) as well as single- or double-strand DNA breaks (31, 93, 94). Although sperm DNA is highly compacted by protamines, certain regions (such as the nuclear matrix attachment regions) remain relatively loose, and sperm rely on limited 8-oxoguanine DNA glycosylase (OGG1) for repair (95). Therefore, oxidative damage easily accumulates and results in SDF. Relevant studies suggest that high SDF significantly increases the risk of male infertility, worsens outcomes of assisted reproductive technologies (e.g., increased miscarriage rates), and may increase the risk of genetic abnormalities in offspring (96, 97). From a clinical evaluation perspective, SDF can often be regarded as one of the comprehensive readout indicators of oxidative stress load, but its upstream driving factors may be multi-sourced, requiring joint interpretation with inflammatory and oxidative stress markers.

**Figure 3:**
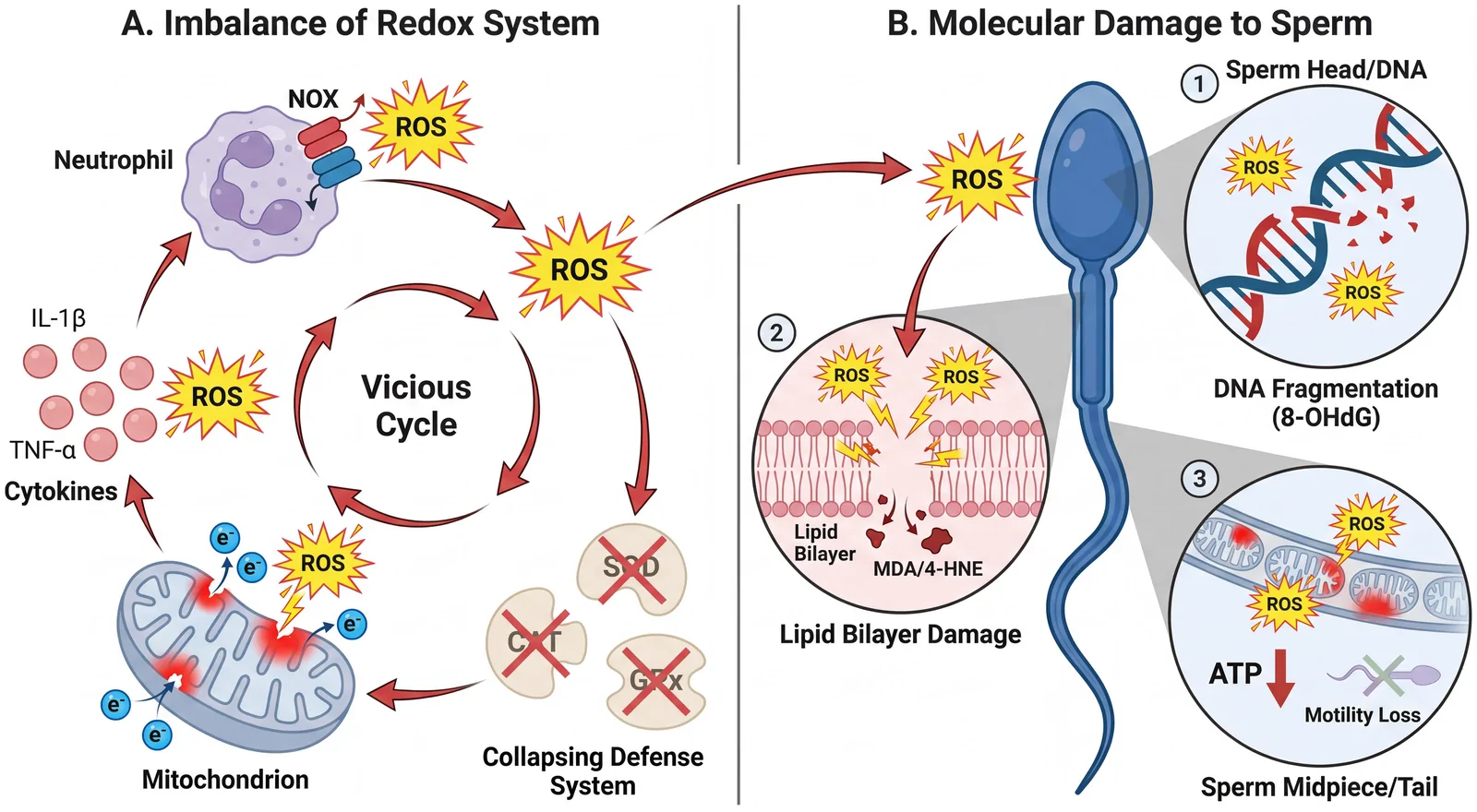
Oxidative-stress cascade in CP/CPPS and its impact on sperm function. **(A)**. Imbalance of the oxidant–antioxidant system: In the CP/CPPS microenvironment, infiltrating inflammatory cells (predominantly neutrophils/macrophages) undergo a “respiratory burst” and generate excessive reactive oxygen species (ROS) mainly via NADPH oxidase (NOX). In parallel, inflammation-induced injury to prostatic/accessory gland epithelial cells triggers mitochondrial dysfunction and electron-transport chain leakage, further amplifying ROS production. Pro-inflammatory cytokines (e.g., IL-1β and TNF-α) can upregulate oxidases including NOX and disrupt mitochondrial homeostasis, reinforcing a pro-inflammatory/oxidative positive-feedback loop. Meanwhile, antioxidant defenses (SOD, CAT, GPx) decline, resulting in persistent ROS accumulation and increased oxidative-damage markers (e.g., MDA and 8-OHdG). **(B)**. Molecular damage to sperm: Due to PUFA-rich membranes and limited cytoplasmic antioxidant reserves, spermatozoa are highly vulnerable to oxidative stress.

Finally, there is protein oxidation and metabolic obstruction. ROS can cause modifications such as carbonylation, sulfhydryl oxidation, or tyrosine nitration in key functional proteins within sperm, leading to structural changes or loss of function (98). This damage is widespread: on one hand, it destroys cytoskeletal structures such as tubulin and dynein, leading to the disintegration of the flagellar motor apparatus and severely weakening sperm motility (99); on the other hand, it inhibits the activity of key metabolic enzymes like creatine kinase and adenylate kinase, blocking the generation of ATP (99–102). ATP is an indispensable energy source for sperm motility, capacitation, and fertilization. Simultaneously, oxidative inactivation of membrane proteins involved in sperm–egg recognition and fusion will directly lead to fertilization failure (98). Therefore, the structural “initiating injury” caused by membrane lipid peroxidation is amplified through metabolism/mitochondria, promoting DNA damage and protein inactivation, ultimately forming a functional collapse chain spanning “motility–capacitation–fertilization.” In summary, oxidative stress, through the triple blow of “membrane lipid peroxidation—DNA fragmentation—protein functional inactivation/metabolic obstruction, “ acts synergistically on the structurally fragile human sperm, constituting the molecular pathological basis of CP-related male fertility decline (**Figure 3****).**

(1) Sperm head/nuclear DNA: ROS (especially hydroxyl radicals) induces oxidative base lesions (8-OHdG) and DNA single- or double-strand breaks, leading to sperm DNA fragmentation (SDF). (2) Plasma-membrane lipid bilayer: ROS attacks membrane PUFAs, driving lipid peroxidation and generating cytotoxic aldehydes such as malondialdehyde (MDA) and 4-hydroxynonenal (4-HNE), which compromise membrane integrity and fluidity and impair motility/fertilization-related functions. (3) Sperm midpiece/tail: Oxidative injury can inhibit metabolic enzymes and block ATP generation; reduced ATP availability contributes to motility decline or loss.

## 5 Disruption of the neuroendocrine network and the local biochemical microenvironment

The pathological microenvironment of Chronic Prostatitis/Chronic Pelvic Pain Syndrome (CP/CPPS) involves not only inflammatory-immune crosstalk and oxidative stress, but alterations in local biochemical components and dysregulation of the neuroendocrine network also represent crucial links in the impairment of sperm development. Studies have revealed a series of local hormone levels and receptor expression patterns that differ significantly from healthy controls. These findings provide a novel perspective and potential targets for elucidating its pathophysiological mechanisms and developing innovative diagnostic and therapeutic strategies (103, 105).

First, the biochemical homeostasis of the prostatic microenvironment constitutes the physicochemical foundation for sperm survival (106). Prostatic fluid, secreted by prostate epithelial cells, is enriched with various enzymes, proteins, and minerals, providing indispensable nutritional support and a protective barrier for spermatozoa. Under physiological conditions, the concentrations of zinc and citrate in prostatic fluid are exceptionally high, reaching hundreds to thousands of times those in blood plasma (approximately 500-fold for zinc and 400–1500-fold for citrate) (107–111). These core physicochemical components play a pivotal role in regulating semen liquefaction, maintaining sperm membrane structural stability, and acting as antioxidants to defend against reactive oxygen species (ROS)-induced damage (112). Second, the collapse of this local biochemical environment is frequently accompanied by significant neuroendocrine regulatory dysregulation. Current evidence indicates that CP/CPPS patients often exhibit characteristic hormonal profile abnormalities, specifically characterized by decreased testosterone levels and relatively elevated estrogen levels resulting from impaired signaling of the hypothalamic-pituitary-gonadal (HPG) axis (103–105). This endocrine disruption does not exist in isolation but is intimately linked to the chronic pain and psychological stress associated with the disease: persistent stress responses can overactivate the hypothalamic-pituitary-adrenal (HPA) axis. The resulting high levels of cortisol not only suppress the pulsatile secretion of gonadotropin-releasing hormone (GnRH) at the hypothalamic level but also directly interfere with the steroidogenic function of testicular Leydig cells (113–117). Thus, the “deterioration of the biochemical environment” caused by prostatic secretory dysfunction intertwines with the “failure of endocrine regulation” triggered by the cross-inhibition of the HPA-HPG axes, collectively forming a complex pathological network that impairs sperm development.

### 5.1 Collapse of the local prostatic biochemical environment: cascading impairment of enzymes, ions, and physicochemical conditions

The biochemical homeostasis of the prostatic microenvironment depends not only on the constancy of local components but is also governed by a precise neuroendocrine network. Biochemically, prostatic fluid secreted by prostate epithelial cells (comprising approximately 20%–30% of total semen volume) forms the physicochemical basis for sperm survival. The enzymes, proteins, and minerals it contains provide indispensable nutritional support and protection for spermatozoa (106). High concentrations of zinc ions, in particular, play a crucial role as a “molecular guardian” in regulating sperm function. On one hand, the highly zinc-enriched microenvironment originating from the prostate participates in the construction of a stable “zinc-sulfur/amino network” through coordination or cross-linking with cysteine- or arginine-rich residues in sperm nucleoproteins and membrane proteins, thereby ensuring the high density of sperm chromatin and the structural stability of the plasma membrane. On the other hand, zinc is not merely an essential cofactor for key antioxidant enzymes such as Cu/Zn-SOD; it effectively prevents energy metabolism imbalances and oxidative stress damage by regulating mitochondrial oxygen consumption and inhibiting premature capacitation and the acrosome reaction (118–120). However, multiple systematic reviews and meta-analyses have confirmed that chronic prostatitis (CP) can lead to significantly impaired prostatic secretory function, primarily manifesting as significantly lower zinc content in the seminal plasma of patients compared to healthy controls, an alteration that has been recognized as a direct marker of prostatic secretory dysfunction (9, 121). Concurrently, the levels of citrate and acid phosphatase (ACP), which serve as critical functional molecules in seminal plasma, also exhibit a significant downward trend under inflammatory conditions (122, 123). This local biochemical imbalance, driven by inflammation, directly precipitates nutritional deprivation and the collapse of the antioxidant barrier in sperm, thereby constructing a direct pathological bridge connecting prostate lesions to sperm functional impairment.

### 5.2 Dysregulation of systemic neuroendocrine axes: crosstalk between the HPA and HPG axes

CP/CPPS is not merely an organ-specific inflammation but a persistent physiological and psychological stressor characterized by chronic pain, anxiety, and depression (124, 125). This chronic stress state continuously activates the body’s core stress response system—the hypothalamic-pituitary-adrenal (HPA) axis—leading to long-term abnormalities in glucocorticoid (mainly cortisol) secretion. Substantial evidence indicates a strong negative “crosstalk” between the HPA axis and the HPG axis (36, 126). Specifically, hyperactivation of the HPA axis can inhibit male reproductive function at a minimum of three levels: First, at the hypothalamic level, stress-induced corticotropin-releasing hormone (CRH) and high levels of cortisol can potently inhibit the pulsatile secretion of gonadotropin-releasing hormone (GnRH), shutting down the “master switch” of the reproductive axis at its source (127). Second, at the pituitary level, cortisol reduces the sensitivity of gonadotrophs to GnRH, leading to decreased secretion of luteinizing hormone (LH) and follicle-stimulating hormone (FSH) (128). Finally, at the testicular level, cortisol can act directly on Leydig cells to inhibit the activity of key enzymes in steroidogenesis, thereby blocking the biosynthesis of testosterone (129).

In this complex neuroendocrine network, abnormal changes in prolactin (PRL) are also a key link. Clinical evidence shows that peripheral blood PRL levels are significantly reduced in CP/CPPS patients, and the degree of reduction is negatively correlated with the severity of erectile dysfunction (ED) (130). Although the physiological regulation of PRL is complex, under pathological conditions, fluctuations in its levels are closely associated with sperm DNA integrity. Research has found that in patients with oligoasthenoteratozoospermia, a high sperm DNA fragmentation index (DFI) is often accompanied by abnormally elevated FSH and LH, and PRL levels are also positively correlated with DFI (131). This suggests that abnormalities in multiple hormone levels, including FSH, LH, and PRL, may act synergistically on the reproductive microenvironment. Therefore, HPA axis dysregulation mediated by CP/CPPS and abnormal changes in hormones such as PRL constitute a potent reproductive inhibition pathway independent of local inflammation. Interwoven with HPG axis dysfunction, they collectively lead to low testosterone levels, spermatogenic arrest, and impaired integrity of genetic material (132).

### 5.3 Functional disorder of the gonadotropic axis and receptor resistance

CP/CPPS can profoundly interfere with the function of the hypothalamic-pituitary-gonadal axis via the neuroendocrine network, leading to abnormalities in the secretory rhythm and actions of gonadotropins. Inflammation-associated cytokine signaling (e.g., IL-1β, TNF-α) can suppress the GnRH drive by interfering with the kisspeptin-GnRH neuronal network, thereby disrupting LH/FSH secretion (particularly destroying the pulsatile rhythm of LH) (133, 134). Furthermore, more direct pathological damage occurs locally within the testes: inflammatory factors (especially TNF-α) can induce the endocytosis and desensitization of FSH receptors, resulting in a marked decrease in the biological responsiveness of testicular tissue to FSH, generating typical “peripheral resistance” (135, 136). FSH signaling is critical for the proliferation and functional maintenance of Sertoli cells: not only does it act synergistically with testosterone to preserve the structural integrity of the blood-testis barrier (BTB), but it also promotes lactate production by Sertoli cells, supplying crucial energy metabolic substrates for spermatogenic cells, thereby safeguarding the metabolic homeostasis of the spermatogenic microenvironment (137–145).

This hormone-driven deprivation of metabolic support inevitably impacts downstream sperm functional components. Multiple studies suggest that mitochondrial function is intrinsically linked to ultimate sperm quality: a decline in mitochondrial membrane potential and insufficient ATP content directly lead to a sharp reduction in sperm motility and fertilization potential, severely affecting acrosome-related functions (146–148). Echoing this mechanism is the significant impairment of acrosin activity. As a pivotal effector molecule for successful fertilization, reduced acrosin activity has been proven to be closely correlated with oxidative stress triggered by local glutathione (GSH) depletion and premature acrosome release (149). This microscopic mechanism aligns highly with clinical findings, wherein large cohort studies of CP/CPPS patients ubiquitously observe a significant decline in total sperm motility and progressive motility (5). Additionally, relevant animal studies suggest that improving androgen-mediated metabolic regulation of accessory sex glands can effectively optimize seminal plasma biochemical composition and significantly enhance sperm mitochondrial function and linear kinematic parameters (VSL, LIN) (150).

### 5.4 Remodeling of the local testicular hormonal microenvironment: inversion of the estrogen/androgen ratio

In the reproductive tract microenvironment of CP/CPPS patients, local hormonal metabolic dysregulation presents a significant imbalance characterized by the “pathological elevation of estrogen” and the “suppression of testosterone synthesis”. Although the increased susceptibility to prostatitis caused by low testosterone and peripheral hormonal changes driven by metabolic syndrome are non-negligible confounding factors, translational medical research has confirmed, after excluding these systemic backgrounds, that the concentration of 17β-estradiol (E2) in the seminal plasma of CP patients is significantly higher than that of healthy controls, whereas peripheral plasma E2 levels show no such variation. This localized phenomenon profoundly underscores the specific remodeling of the local endocrine microenvironment by inflammation: (104). Under physiological conditions, estrogen precisely regulates spermatogenesis and maintains the integrity of the blood-testis barrier (BTB) via its receptors (ERα, ERβ, GPER) and epigenetic modifications (151–153). However, in local tissues, steroid metabolic reprogramming is driven by pro-inflammatory factors such as TNF-α: inflammatory cytokines induce the overexpression of aromatase (Aromatase/CYP19A1), accelerating the conversion of the substrate testosterone into estradiol and leading to an excessive local production of E2 (154, 155). This hyperestrogenic state is frequently accompanied by the downregulation of androgen receptor (AR) expression, causing a severe imbalance in the estrogen/androgen receptor (ER/AR) ratio (103, 105), which subsequently disrupts Sertoli cell function and ultimately inhibits sperm development and capacitation (104). In sharp contrast to elevated estrogen, and carrying even graver consequences, is the profound suppression of testosterone synthesis and its signaling pathways. Studies have demonstrated that under persistent inflammatory conditions, Leydig cells can become direct targets of inflammatory factors: TNF-α can inhibit the expression of Cyp17/P450c17 and reduce cAMP-stimulated testosterone production; furthermore, while inhibiting Leydig cell differentiation and regeneration, IL-6 downregulates the expression of key steroidogenic genes such as Star, Cyp11a1 (P450scc), and Cyp17a1, thereby severely attenuating testosterone biosynthesis (52, 154, 155).

This local synthesis obstacle can trigger significant adverse consequences. Under physiological states, the testosterone associated with the testes and seminiferous tubules (intratesticular testosterone, ITT) is maintained at levels substantially higher than in peripheral blood—typically 25 to 125 times that of serum. This exceptionally high concentration is considered a critical prerequisite for ensuring adequate activation of AR signaling in Sertoli cells, thereby safeguarding normal spermatogenesis, particularly pivotal stages such as meiosis and sperm release (156–158). When inflammation causes ITT levels to plummet below this threshold, a chain reaction of pathology is initiated: first, the blood-testis barrier (BTB) loses its dynamic remodeling capacity, and tight junction proteins disassemble, preventing germ cells from successfully migrating across the barrier toward the luminal side (158–161); second, the meiotic progression of spermatocytes is forcibly halted, and cell cycle arrest induces massive apoptosis of germ cells (162–165); simultaneously, the ectoplasmic specialization (adhesion junctions) between Sertoli cells and spermatids weakens, leading to the premature sloughing of immature spermatozoa (156). Furthermore, this testosterone deprivation effect cascades downstream, destroying the androgen-dependent microenvironment of the epididymis and causing biochemical composition changes in the sperm membrane, which severely impedes the completion of their final maturation and capacitation processes (56, 61, 166).

**Figure 4:**
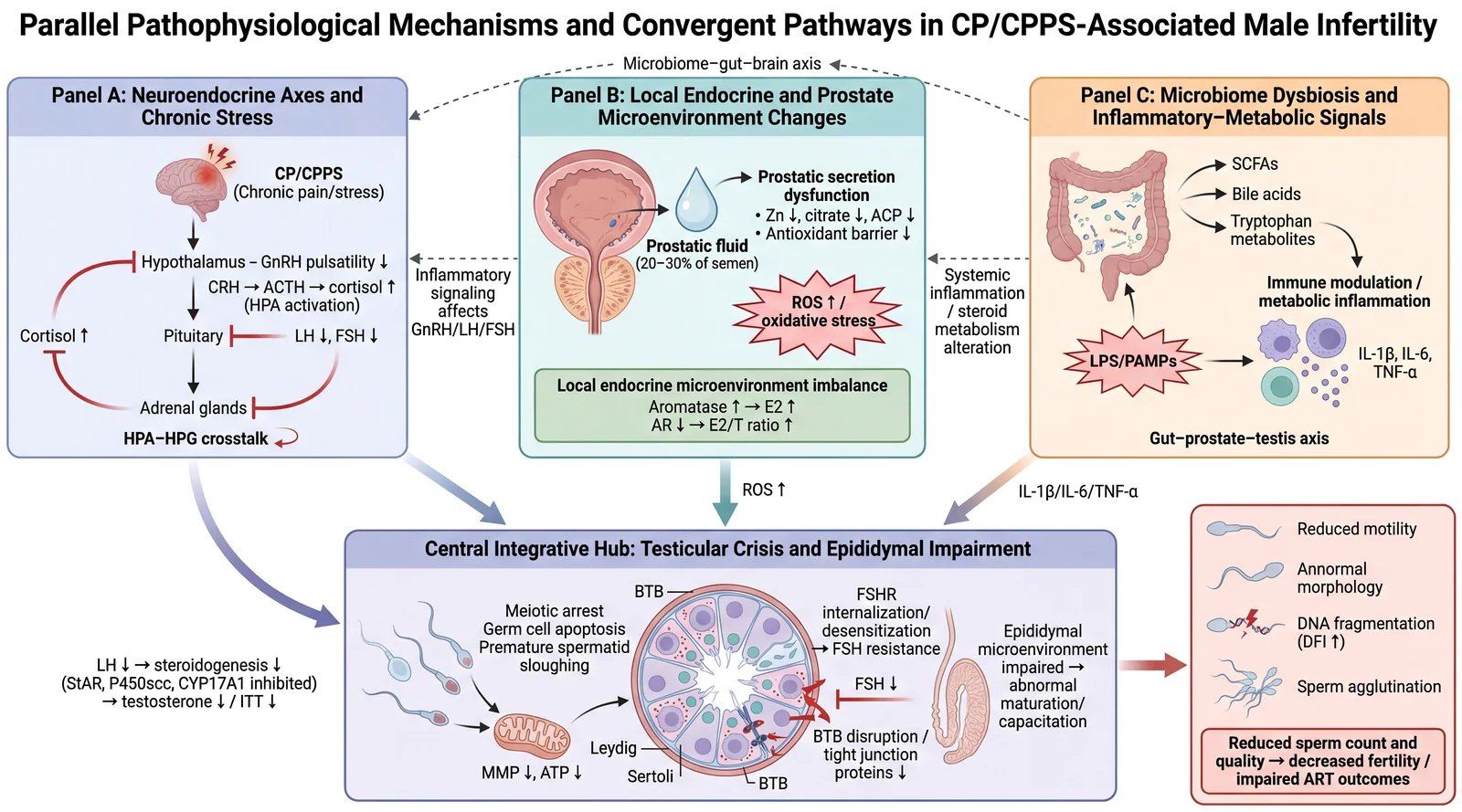
Synergistic mechanisms of neuroendocrine, microenvironmental, and microbiome dysregulation in CP/CPPS-induced sperm impairment. **(A)** Neuroendocrine Dysregulation: Chronic pain stress activates the HPA axis (Cortisol ↑), which suppresses hypothalamic GnRH pulsatility and pituitary gonadotropin secretion (LH/FSH ↓) via HPA-HPG axis crosstalk. **(B)** Local Biochemical & Endocrine Alterations: Prostatic dysfunction depletes key nutrients (Zn, Citrate) and antioxidant barriers, promoting oxidative stress. Concurrently, increased Aromatase activity and downregulated Androgen Receptor (AR) lead to a pathological inversion of the E2/T ratio. **(C)** Microbiome-Immune Interaction: Dysbiosis of the gut-genitourinary microbiota modulates the “Gut-Prostate-Testis Axis” through altered metabolites (e.g., SCFAs) and LPS-mediated immune activation, triggering the release of pro-inflammatory cytokines (IL-1β, TNF-α). These pathways converge on the testicular-epididymal microenvironment to: (1) inhibit steroidogenic enzymes (StAR, P450scc, CYP17A1), blocking Testosterone/ITT synthesis; (2) induce FSH receptor (FSHR) desensitization and Blood-Testis Barrier (BTB) disruption; and (3) impair mitochondrial membrane potential (MMP) and ATP production. Collectively, this results in increased sperm DNA fragmentation (DFI), motility loss, and abnormal morphology.

In summary, by inducing local aromatase overexpression and directly inhibiting key testosterone-synthesizing enzymes, CP/CPPS establishes a pathological endocrine microenvironment of “high estrogen, low androgen” within the reproductive tract. This severe inversion of the estrogen/androgen ratio not only cuts off the supply of extremely high concentrations of testosterone necessary for spermatogenesis at its source—leading to BTB collapse, meiotic arrest, and massive apoptotic depletion of spermatogenic cells—but also inflicts a secondary hit on the acquisition of sperm function by interfering with the downstream androgen-dependent maturation process in the epididymis. Therefore, local hormonal imbalance constitutes an independent and core endocrine pathological axis by which CP/CPPS impairs male fertility. It deeply intertwines with the aforementioned mechanisms of immune inflammation and oxidative stress, ultimately collectively exacerbating the comprehensive disruption of the spermatogenic microenvironment (**Figure 4**).

## 6 The Microbiome: another key regulator of sperm development in CP

The role of the human microbiome in health and disease has garnered widespread attention in recent years. Accumulating evidence indicates that gut and other mucosal-associated microbiota are closely related to, and potentially participate in, the development of various chronic inflammatory and autoimmune diseases (e.g., rheumatoid arthritis, Hashimoto’s thyroiditis) via pathways such as immune regulation and metabolic remodeling mediated by microbial metabolites (167–170). Correspondingly, the genitourinary system is increasingly viewed as an “immune–endocrine–barrier” complex that is highly dependent on local and distal microecological homeostasis.

Current understanding of CP/CPPS has shifted from the traditional “single pathogen infection” model to a more complex “dysbiosis” model. Epidemiological and immunological studies suggest that CP/CPPS may be a disease characterized primarily by chronic abacterial inflammation with autoimmune involvement, and its development is closely related to dysbiosis of the gut, urinary, and reproductive tract microbiota (171–173). Historically, the male reproductive tract was viewed as a “sterile compartment, “ but modern high-throughput sequencing technologies have revealed that semen and prostatic fluid harbor unique low-biomass microbial communities. Although their exact origins and colonization mechanisms remain to be elucidated, their homeostasis is crucial for reproductive health (174, 175).

In patients with CP/CPPS (particularly NIH types III and IV), clinical studies based on 16S rRNA and metagenomic sequencing have confirmed that the homeostasis of the genitourinary microecosystem is disrupted, presenting a characteristic pattern of “depletion of protective flora and enrichment of pathogenic flora.” Specifically, in patient semen or prostatic fluid samples, the abundance of Lactobacillus species, which possess probiotic and anti-inflammatory properties, is significantly decreased, while the abundance of opportunistic pathogens represented by the phylum Proteobacteria (e.g., Pseudomonas, Escherichia) and Streptococcus is significantly increased (176, 177).

Clinical correlation analyses further reveal a high degree of consistency between this microecological dysbiosis and the deterioration of sperm quality. In the semen of healthy males, microbial communities dominated by Lactobacillus iners (L. iners) often correspond to superior semen parameters; conversely, in patients with CP/CPPS, a depletion of Lactobacillus, or the colonization by Neisseria species and Klebsiella pneumoniae, is closely associated with seminal hyperviscosity and oligoasthenoteratozoospermia (174, 178–180). Further research has expanded the scope to the “gut–prostate–reproductive axis.” Multi-center cohort studies and genetic causal inference analyses suggest a correlation, and potentially a causal link, between gut microbiota dysbiosis and susceptibility to prostatitis; concurrently, multiple clinical studies have observed a trend correlating the severity of dysbiosis with declines in sperm concentration, motility, and normal morphology rates (5, 172, 173). Synthesizing the latest evidence regarding the “gut–brain–prostate axis” and the “gut–testis axis, “ the dysregulation of the reproductive tract and gut microbiomes can be regarded as one of the core mechanisms driving the pathological progression of CP/CPPS and impairing male fertility (181).

### 6.1 Multidimensional mechanisms of microbiome-induced sperm impairment

The role of the microbiota in CP/CPPS-related male infertility acts not through a single pathway but by constructing a complex damage network encompassing physical contact, immune activation, oxidative stress, and metabolic interference.

First, direct microbe–sperm/gonad interaction. *In vitro* experiments suggest that when human semen/sperm are co-incubated with Gram-negative urogenital pathogens, bacterial adhesion occurs, inducing sperm agglutination and limiting flagellar motion. Furthermore, in whole semen systems, live bacteria exposure can further trigger enhanced mitochondrial oxidative stress, caspase activation, and increased sperm DNA fragmentation via direct contact or secreted factors (182). Consistent with this, Sahnoun et al. demonstrated that lipopolysaccharides (LPS) from Escherichia coli can significantly reduce sperm motility and viability *in vitro* (183).

Second, immune activation and the systemic impact of the “gut-prostate-testis axis” (primarily based on animal models). PAMPs released by dysbiotic flora activate the TLR2/4–MyD88 pathway. Regarding how gut-derived pathogenic factors affect the prostate “cross-organ, “ Experimental Autoimmune Prostatitis (EAP) mouse models provide key evidence: gut dysbiosis can drive the differentiation of Th17 cells, which then migrate to the prostate, triggering autoimmune inflammation (181, 184–186). Although correlations between systemic inflammatory markers and gut dysbiosis are observed in clinical patients, direct histological evidence of bacterial translocation is difficult to obtain in humans. However, this immune cascade has been confirmed in animal models to disrupt the BTB, inducing autoimmune orchitis and epididymitis (59, 187–190).

Third, inflammation-induced oxidative stress amplification. At the clinical observational level, cohort studies based on reproductive tract infections and microbiome abnormalities reveal a significant “inflammation-oxidation-injury” co-occurrence pattern. Specifically, the detection of pathogens like Ureaplasma urealyticum and Mycoplasma hominis often overlaps with an inflammatory phenotype in semen (leukocytospermia) (191, 192). Further analysis suggests an intrinsic link between Ureaplasma urealyticum infection and elevated semen ROS/MDA levels and DFI (193). Mechanistic studies provide molecular explanations: bacterial products can stimulate leukocytes to undergo a respiratory burst, releasing massive amounts of ROS (187, 194). Furthermore, research on neutrophil “respiratory burst” confirms that bacteriospermia is a strong inducer of oxidative stress (195). However, it must be objectively reviewed that the above evidence mostly originates from generalized reproductive tract infection or infertility cohorts and *in vitro* models; the longitudinal causal chain specific to CP/CPPS populations remains to be completed.

Fourth, metabolic remodeling by microbial metabolites. In animal models, researchers found that as probiotics (like Lactobacillus) decrease, the production of beneficial short-chain fatty acids (SCFAs) such as butyrate decreases (196). SCFAs are critical for maintaining the epithelial barrier and immune regulation; their deficiency may exacerbate systemic and local inflammation. Furthermore, experimental evidence suggests that gut dysbiosis may affect host testosterone metabolism (173, 197). Certain gut and reproductive tract bacteria may also participate in the metabolism of steroid hormones (198–201). Although these metabolic mechanisms have emerged in animal models, their exact weight in human CP/CPPS pathology awaits further quantification. In summary, microbiome dysbiosis participates in CP/CPPS-related male fertility impairment through these four interconnected pathways.

## 7 Intervention strategies and translational research based on microenvironment remodeling

Given the intimate link between the local inflammatory–oxidative stress microenvironment of CP/CPPS and seminal quality abnormalities, recent years have witnessed a surge in research focusing on anti-inflammatory, antioxidant, endocrine correction, and microecological interventions. Although current guidelines prioritize multimodal therapies based on phenotypic stratification, with pharmacological interventions primarily aimed at alleviating pain and lower urinary tract symptoms (LUTS) (202), select high-quality clinical studies have begun to explore the potential of “targeted microenvironment regulation strategies, “ providing critical evidence for their feasibility. For instance, in a population with chronic bacterial prostatitis (NIH Type II), Cai et al. conducted a prospective randomized controlled trial (RCT) to evaluate the efficacy of antibiotics combined with Flogofilm^®^, an antioxidant and anti-inflammatory phytotherapeutic agent rich in N-acetylcysteine and resveratrol. The results demonstrated that, compared to antibiotic monotherapy, the combination therapy group exhibited a significantly greater reduction in total IPSS and NIH-CPSI scores over a 6-month follow-up period (203). This finding strongly suggests that adjuvant intervention strategies aimed at improving the microenvironment (anti-inflammatory/antioxidant), when added to conventional anti-infective therapy, can more effectively reverse local prostatic pathology and improve clinical symptoms. However, it must be acknowledged that existing evidence, including the aforementioned RCT, still primarily utilizes symptom improvement as the primary endpoint, without directly incorporating assessments of semen parameters, sperm DNA fragmentation index (DFI), or pregnancy/live birth outcomes. Moreover, as this evidence is derived from NIH Type II cases, caution must be exercised when extrapolating these findings to NIH Type III patients. This evidential gap—where “symptom improvement” is confirmed but “reproductive benefit” remains unknown—clearly defines the critical breakthrough direction for future translational research: there is an urgent need to establish a multidimensional evaluation system integrating “objective microenvironmental markers (e.g., seminal plasma inflammatory cytokines/oxidative stress markers)—semen quality/DFI—reproductive outcomes.” This is essential to definitively validate the clinical value of microenvironment remodeling interventions in preserving male fertility.

From a clinical translational perspective, interventions related to microenvironment remodeling are most appropriately implemented and evaluated based on “phenotypic stratification.” This involves classifying patients into subtypes such as **inflammation-dominant** (elevated inflammatory factors in seminal plasma/prostatic fluid, NIH-IIIa tendency), **oxidative stress-dominant** (elevated ROS/decreased antioxidant capacity), **endocrine imbalance-dominant** (abnormal Testosterone or T/E2 ratio, HPA–HPG axis disturbance), and **dysbiosis-dominant** (abnormalities in microbiota diversity/metabolite profiles). Correspondingly, in addition to symptom-based endpoints like the NIH-CPSI and IPSS, it is recommended to simultaneously incorporate stratified endpoints covering “objective microenvironmental markers—semen quality/function markers—reproductive outcomes.” These include seminal plasma inflammatory cytokine profiles, oxidative stress indices (e.g., TAC/ROS), hormonal axis indicators (e.g., T, T/E2), and conventional semen parameters along with DFI. Based on this stratification framework, a mapping relationship of “mechanism target—biomarker—intervention choice” can be established, providing an actionable roadmap for the precision of multi-target combination therapies.

Currently, anti-inflammatory and antioxidant strategies remain the cornerstone of interventions targeting the pathological remodeling of the local microenvironment. Regarding anti-inflammatory therapy, COX-2 inhibitors (e.g., rofecoxib, celecoxib) have demonstrated potential benefits in reducing symptom scores in randomized trials, and inflammatory markers in prostatic fluid or seminal plasma (e.g., IL-8) can serve as objective supplements for efficacy monitoring (60, 204, 205). However, reproductive safety warrants caution; certain NSAIDs (e.g., ibuprofen) may interfere with testicular endocrine homeostasis, leading to “compensated hypogonadism, “ necessitating a careful risk-benefit assessment for men attempting conception (206). Since inflammation often induces oxidative stress cascades, antioxidant and metabolic support interventions are viewed as potential synergistic strategies. Systematic reviews and meta-analyses of relevant RCTs suggest that supplements such as L-carnitine (or acetyl-L-carnitine) and Coenzyme Q10 can improve semen parameters (e.g., sperm motility, concentration, or morphology) in select male infertility populations (207, 208). An RCT involving 350 patients confirmed that a combination regimen containing L-carnitine significantly improved sperm motility and morphology (209). In preclinical studies, interventions targeting the NLRP3 inflammasome–oxidative stress axis have shown translational potential; candidate agents such as lycopene and melatonin have been confirmed to simultaneously alleviate inflammatory injury and oxidative stress responses in animal models via signaling networks like NF-κB/MAPK and Nrf2 (210, 211). However, the translation of these findings to clinical reproductive outcomes awaits confirmation through multi-center studies.

Beyond local interventions, endocrine regulation and microecological reconstruction provide systemic therapeutic perspectives. When patients present with concurrent HPG axis dysfunction, the use of SERMs (e.g., Clomiphene) or aromatase inhibitors (e.g., Anastrozole) can elevate serum testosterone and potentially improve semen parameters. Although EAU guidelines maintain a cautious stance on idiopathic infertility, the AUA/ASRM suggests these as off-label options when specific hormonal ratios are abnormal (212–216). Concurrently, microecological interventions are receiving increasing attention. Clinical evidence indicates that probiotics or synbiotics contribute to improving semen quality and reducing systemic inflammation levels (217, 218). Mechanistic studies further reveal that gut-derived metabolites (such as butyrate) can alleviate prostatic inflammation by regulating the “microbiota–immune–oxidative stress” axis, offering new insights for interventions based on “postbiotics” (219). In summary, although evidence for single intervention strategies varies, given the complexity of CP/CPPS pathology, the most feasible future direction lies in constructing multi-target combination intervention regimens covering “anti-inflammation + antioxidation + endocrine correction + microecological regulation” based on patient phenotypic stratification, with the aim of achieving the dual goals of symptom relief and fertility protection (**Table 1**).

**Table 1:** Evidence for interventions targeting chronic prostatitis–associated microenvironment remodeling and sperm developmental impairment: key mechanisms and translational overview.

Domain	Representative interventions	Mechanistic rationale	Key outcomes/biomarkers	Limitations	Refs.
Anti-inflammatory/immunomodulatory	COX-2 inhibitors (celecoxib/rofecoxib); NSAIDs (caution in men trying to conceive)	Suppress COX-2–mediated inflammation	NIH-CPSI↓; inflammatory markers↓ in seminal plasma/prostatic fluid (e.g., IL-8)	Primarily symptom endpoints; reproductive safety should be weighed in preconception men	(60, 204–206)
Antioxidant/metabolic support	L-carnitine, CoQ10; lycopene/melatonin	Reduce ROS burden; modulate NLRP3/Nrf2-related pathways	Improved sperm motility/morphology; ↓oxidative-stress indices/↑antioxidant capacity in semen	nfertile cohort bias;Varied doses/formulations; Unproven clinical outcomes	(207, 208, 210, 211)
Endocrine correction	Clomiphene; anastrozole	Boost endogenous testosterone and restore T/E2 balance	↑serum T and T/E2; possible improvement in semen parameters (concentration, total count, motility)	Applicable only to hormonal-abnormal subgroups; partly off-label; requires close monitoring and long-term follow-up	(212–216)
Microbiome-targeted intervention	Probiotics/synbiotics; butyrate/postbiotic-inspired strategies	Modulate the “microbiota–immunity–oxidative stress” axis	Trend toward improved semen quality; ↓systemic inflammatory markers	Heterogeneity in strains/dose/duration; limited causal evidence and standardization	(217–219)
Combination therapy	Antibiotics + anti-inflammatory/antioxidant nutraceuticals (e.g., Flogofilm; NAC/resveratrol, etc.)	Multi-target synergy	Improved IPSS/NIH-CPSI; (semen/DFI/pregnancy outcomes not assessed)	Open-label/No placebo;Small sample/Short follow-up;Subjective endpoints (Symptom-driven);No semen/fertility data;Confounding multi-ingredients	(203)

## 8 Conclusions and future perspectives

In summary, CP/CPPS is not merely a simple localized infection, but rather a complex pathological microenvironment interwoven by an “immune-inflammatory storm, oxidative stress imbalance, neuroendocrine network dysregulation, and microbial heterogeneity.” This microenvironment exerts profound cascading toxic effects on the entire life cycle of spermatozoa, comprehensively covering critical stages from spermatogenesis and epididymal maturation to fertilization and capacitation. Although the individual mechanisms of these four major dimensions have gradually become clearer, their spatiotemporal interaction networks across organs (e.g., the gut-prostate-testis axis) and systems (e.g., the HPA-HPG axis) remain far from being fully elucidated. In the future, basic research urgently needs to leverage cutting-edge technologies, such as single-cell sequencing and spatial multi-omics, to deeply map the intercellular communication networks of these master regulatory loops. At the clinical translation level, it is imperative to break through current diagnostic and therapeutic bottlenecks and translate these mechanistic breakthroughs into actionable precision intervention strategies. Based on this, we propose the following recommendations for future research.

The transition towards precision microenvironment stratification and liquid biopsy paradigms is crucial. A major clinical pain point is that traditional symptom-centric evaluation systems (such as the NIH classification) fail to accurately reflect the degree of impairment in the reproductive tract microenvironment. Therefore, clinical management urgently needs to pivot towards a mechanism-based stratification strategy oriented towards fertility protection and reproductive outcomes. By integrating seminal plasma biochemical profiles, immune-metabolic signatures, and sperm genetic integrity (e.g., DFI), a “non-invasive liquid biopsy” system with high reproducibility and follow-up value can be established. This transition will propel the diagnosis and treatment of CP/CPPS from an empirical “one-size-fits-all” approach to precision medicine driven by microenvironmental characteristics. For instance, by quantitatively measuring oxidative damage markers (e.g., 8-OHdG) or core inflammatory cytokines (e.g., IL-8, TNF-$\alpha$) in seminal plasma, patients can be precisely subtyped into “high oxidative stress” or “high inflammation” phenotypes. This provides direct evidence for subsequent targeted antioxidant or anti-inflammatory interventions, comprehensively extending this stratification logic to systemic endocrine and microecological interventions.

To make precision stratification truly clinically actionable, it is necessary to construct executable “endophenotype” monitoring panels. Multidimensional pathological mechanisms should be mapped into a standardized “seminal liquid biopsy” combination panel. This panel must comprehensively encompass: (i) inflammatory/immune markers (cytokine/chemokine profiles, AsAb, etc.); (ii) oxidative stress and damage indicators (ROS/TAC imbalance status, MDA, 8-OHdG, etc.); (iii) sperm function and genetic integrity metrics (routine semen parameters and DFI/chromatin condensation abnormalities); and (iv) endocrine and accessory sex gland biochemical surrogate markers (e.g., local T/E$_2$ ratio, zinc, citrate). When feasible, microbiome and related metabolite clues (e.g., LPS/PAMP exposure, SCFA abundance) should be supplemented. The core objective of incorporating these multidimensional metrics is no longer limited to merely describing the disease state, but rather to define “endophenotypes” with high clinical guiding significance (e.g., immune-activated, oxidative-damaged, endocrine-disrupted, and microecologically-imbalanced phenotypes). This framework will support the risk stratification of high-risk populations at baseline and replace subjective symptom scores with objective biological responses during treatment, enabling the dynamic monitoring of targeted therapies.

Ultimately, this precision stratification system must guide targeted therapies and reshape clinical trial endpoints. Based on distinct endophenotypes, clinicians can develop individualized intervention strategies: prioritizing anti-inflammatory and immunomodulatory therapies for high-inflammation phenotypes with IL-8 reductions as pharmacodynamic readouts; enhancing antioxidant and metabolic support for high-oxidative-damage phenotypes to establish a mechanistic closed-loop from microenvironmental correction to genetic integrity improvement; implementing endocrine correction (e.g., SERMs) for those with significant endocrine abnormalities under strict indications, utilizing hormone axis metrics as core mechanistic endpoints; and exploring regulatory strategies targeting the “microbiome-immune-oxidative” axis for microecological imbalance phenotypes. Overall, the most promising translational direction is the construction of a multi-target combination regimen based on endophenotypes, encompassing “anti-inflammation + antioxidation + endocrine correction + microecological reconstruction.” Furthermore, clinical trials aimed at reproductive outcomes must fundamentally reshape their evaluation endpoints to form a hard-outcome closed-loop validation comprising symptom scales, objective microenvironment biomarkers, semen quality/function (including DFI), and clinical pregnancy/live birth rates. Simultaneously, studies should strengthen inclusion and exclusion criteria and differential diagnosis workflows to maximally isolate confounding factors within the disease spectrum, truly achieving the leap from basic mechanistic elucidation to clinical fertility benefits.
