# Correction: Research progress on the multidimensional mechanisms underlying impaired spermatogenesis induced by pathological microenvironment remodeling in chronic prostatitis

**DOI:** 10.3389/fimmu.2026.1981675

**Published:** 2026-09-08

**Authors:** Jing Li, Zhipeng Jiang, Wen Luo, Kaihua Tang, Decan Liang, Xijian Luo, Lei Liu, Zongmin Long, Hui Huang, Weiwei Chen, Yichi Zhang

**Affiliations:** Department of Urology, The Third Affiliated Hospital of Zunyi Medical University (The First People’s Hospital of Zunyi), Zunyi, China

**Keywords:** chronic prostatitis, immune cells, microenvironment, oxidative stress, sperm, sperm DNA fragmentation

An incorrect **Funding** statement was provided. The correct **Funding** statement reads:

“The author(s) declared that financial support was received for this work and/or its publication. This research was supported by grants from the Department of Science and Technology of Guizhou Province [grant number: ZK (2023) 484], the Zunyi City Science and Technology Program (grant numbers: HZ (2023) 467; HZ (2024) 20), the Guizhou Provincial Basic Research Program (General Project) (Qiankehe Basics-MS[2026]871), and the Beijing Dadi Medical Charity Foundation [grant number: 22025H18].”

The original version of this article has been updated.

